# Flexible Temperature Sensor Integration into E-Textiles Using Different Industrial Yarn Fabrication Processes

**DOI:** 10.3390/s20010073

**Published:** 2019-12-21

**Authors:** Pasindu Lugoda, Julio C. Costa, Carlos Oliveira, Leonardo A. Garcia-Garcia, Sanjula D. Wickramasinghe, Arash Pouryazdan, Daniel Roggen, Tilak Dias, Niko Münzenrieder

**Affiliations:** 1 Sensor Technology Research Centre, University of Sussex Falmer, Brighton BN1 9QT, UK; jc711@sussex.ac.uk (J.C.C.); l.a.garcia-garcia@sussex.ac.uk (L.A.G.-G.); a.pouryazdan@sussex.ac.uk (A.P.); daniel.roggen@ieee.org (D.R.); n.s.munzenrieder@sussex.ac.uk (N.M.); 2 Advanced Textiles Research Group, Nottingham Trent University Nottingham NG1 4GG, UK; jose.oliveira@ntu.ac.uk (C.O.); tilak.dias@ntu.ac.uk (T.D.); 3 Stretchline (Zhongshan) Limited, Goldenbell Section, Fu Zhong Lu, Shunjing Industrial Park, Banfu Town, Zhongshan City 528459, China; sanjulaw@stretchlinehk.com; 4 Faculty of Science and Technology, Free University of Bozen-Bolzano, 39100 Bozen, Italy

**Keywords:** electronic textiles, E-textiles, flexible electronics, wearable electronics, smart textiles, temperature sensing, resistance temperature detectors (RTD), sensor integration

## Abstract

Textiles enhanced with thin-film flexible sensors are well-suited for unobtrusive monitoring of skin parameters due to the sensors’ high conformability. These sensors can be damaged if they are attached to the surface of the textile, also affecting the textiles’ aesthetics and feel. We investigate the effect of embedding flexible temperature sensors within textile yarns, which adds a layer of protection to the sensor. Industrial yarn manufacturing techniques including knit braiding, braiding, and double covering were utilised to identify an appropriate incorporation technique. The thermal time constants recorded by all three sensing yarns was <10 s. Simultaneously, effective sensitivity only decreased by a maximum of 14% compared to the uncovered sensor. This is due to the sensor being positioned within the yarn instead of being in direct contact with the measured surface. These sensor yarns were not affected by bending and produced repeatable measurements. The double covering method was observed to have the least impact on the sensors’ performance due to the yarn’s smaller dimensions. Finally, a sensing yarn was incorporated in an armband and used to measure changes in skin temperature. The demonstrated textile integration techniques for flexible sensors using industrial yarn manufacturing processes enable large-scale smart textile fabrication.

## 1. Introduction

Unobtrusive continuous monitoring of skin temperature plays an important role in many facets of medicine [1,2,3,4,5,6,7,8,9,10] and sports [11,12,13]. Temperature is an established marker to detect infections in wounds [3,4] and can be used to predict the occurrence of diabetic foot ulcers [5,6]. Temperature is also used to monitor health in infants [7] and to detect thermal discomfort within a prosthetic socket [8,9,10]. In prosthetics, monitoring and predicting the residual limb skin temperature is regarded as important since the socket of the prosthesis creates a warm and humid microenvironment that encourages growth of bacteria which lead to skin breakdown [8,9,10]. Localised skin temperature monitoring, utilising sensors that remain concealed within everyday textile garments, could therefore greatly benefit patients and healthcare personnel. This can be achieved by embedding temperature sensors within textile yarns. Textile yarns are strands of materials used to manufacture textiles. This method of incorporating sensors within textile yarns provides several advantages. First, the sensors are not visible on either face of the resulting fabric. Second, the temperature sensor fabrics can be produced using conventional textile equipment. Third, the required textile characteristics of softness, comfort, and conformability are not compromised by the integration of sensors. Although numerous flexible temperature sensors are available in the market, most of these sensors have not been integrated in a concealed manner. Moreover, they do not exhibit textile characteristics such as the ability to bend, drape, or shear [14,15,16,17,18].

An ideal approach to fabricate sensing yarns would be to embed thin and fully flexible sensors within the core of textile yarns. Thin-film technology can be utilised to fabricate ultraflexible sensors that can be rolled or folded without altering the sensors’ performance [19]. In addition, thin-film fabrication processes offer numerous advantages such as low cost, large area compatibility, and high scalability [20,21]. Flexible resistive temperature sensors have been fabricated by patterning gold on polyimide [22]. These sensors were previously integrated into woven fabrics, however, this method of integration does not completely conceal the sensors nor protect them from the external environment [23]. Therefore, these sensors affect the aesthetics of the textile and can be easily damaged when worn. Thin-film temperature sensors were also patterned on the surface of nylon yarns [24]. However, so far, the ability of these yarns to undergo bending has not been reported.

Textile structures integrated with metal wires or conductive threads have been used to create temperature sensors which exhibit textile characteristics [25,26,27]. However, since the wires and conductive threads are visible on the surface of the textile, the aesthetic is affected. In addition, these sensors were affected by hysteresis and were unable to provide localised temperature measurements due to their large topology. Rigid surface mount temperature sensors were previously embedded within textile yarns [28,29,30]. The thickness of these yarns has been in the range of 2 mm, which significantly adds to the thickness of the resulting textile knitted or woven using these yarns [28]. Furthermore, rigid chips and large yarn diameters reduce the comfort level of the textile. Researchers have incorporated flexible polyimide strips with bare die thermistors in textile yarns, however, the width of the resulting yarns was >3 mm [31,32]. The incorporation of fully flexible thin-film sensors within the yarn could greatly reduce the yarn size. This may improve the aesthetics, comfort level, and reduce the thickness of the resulting textiles knitted or woven using these yarns.

This work investigates the effects of embedding flexible resistance temperature detectors (RTD) within the fibres of a textile yarn. The fibre cover adds a layer of protection to the sensor and conceals it from the wearer. Three different methods of covering were used to embed the RTDs, namely, knit braiding, braiding, and double covering. The effects on the sensors as a result of the different covering techniques have been investigated. Finally, a braided sensing yarn was integrated into an armband that was knitted using an industrial knitting machine. This armband was utilised to unobtrusively capture changes in skin temperature as a result of physical activity.

## 2. Materials and Methods

This section presents the fabrication procedures of the three different flexible temperature-sensing yarns and the flexible RTD. The techniques utilised to characterise these yarns is also explained here. Finally, it presents the integration of a sensing yarn into a knitted armband to monitor changes in skin temperature.

### 2.1. Fabrication of the Flexible Resistance Temperature Detectors

**Figure 1:**
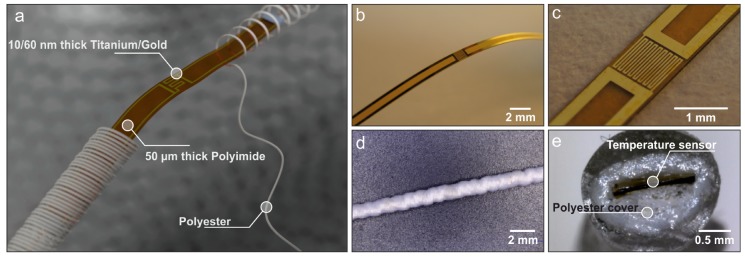
(**a**) Concept of the flexible temperature sensor embedded within the fibres of a textile yarn. (**b**) Bending of the uncovered flexible resistance temperature detectors (RTD). (**c**) Close-up of the sensing area of the RTD. (**d**) RTD embedded within a braided polyester yarn. (**e**) Cross section of the braided temperature-sensing yarn (Epoxy resin was utilised to harden the yarn in order to produce a clean cut).

Temperature sensors were fabricated on a 50 μm thick polyimide substrate. The individual sensors were 75 mm long and 1 mm wide. This elongated form factor was designed to optimise the yarn embedding process. The active layer of these devices consists of 10/60 nm Ti/Au. Ti was deposited to improve the adhesion of Au to the polyimide substrate, whereas Au was used as the thermally sensitive layer. Both materials were deposited through e-beam evaporation and patterned using lift-off. The devices were passivated with a 2 μm thick SU-8 blanket layer deposited through spin-coating. Following their fabrication, the sensors were separated from the main substrate using a dicing saw. A flexible sensor strip is displayed in Figure 1b,c, while Figure 1a shows a representation of the final sensors embedded in the yarns.

### 2.2. Construction of the Temperature-Sensing Yarn

Yarns are the basic blocks in the fabrication of textiles. Flexible sensors embedded within the textile yarns ensure unobtrusive and seamless integration of the sensors within the resulting textile. In this work, the prototype yarns were created utilising three different yarn fabrication techniques: knit braiding, braiding, and covering. The first step of the integration process was to solder 0.15 mm enamelled copper wires (FD Sims Ltd, Blackburn, UK) onto the solder pads of the RTD. The soldering process was completed using a Thermoflo heat gun. Thereafter, the solder joint was secured using a 2-component Epoxy resin. A 357 dtex 2x end 1-ply acrylic (Uppingham yarns, Uppingham, UK) carrier yarn was then attached to the edges of the polyimide strip using the 2-component adhesive. The carrier yarn provided additional mechanical strength to the flexible strip. The enamelled copper wires were wrapped around the carrier yarn. This protects the copper wires and the solder joints from mechanical strain to which the temperature-sensing yarn would be subjected to during the fabric manufacturing process.

**Figure 2:**
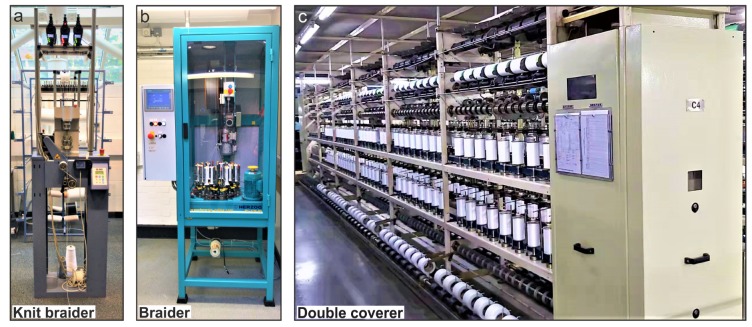
Industial machines utilised for covering the flexible RTDs. (**a**) Rius MC/2 knit braider from RIUS-COMATEX used to create the knit braided yarn. (**b**) RU 1/24-80 braiding machine from Herzog^®^ utilised to fabricate the braided yarn. (**c**) Menegatto 1500 (Menegatto S.r.L) used to build the double covered yarn.

The final step to create a temperature-sensing yarn was to cover the flexible strip, interconnects, and the carrier yarn using a fibre sheath. Three different industrial yarn manufacturing techniques were utilised for covering the flexible RTDs. The first method was undertaken utilising a Rius MC/2 knit braider (Rius-Comatex, Barcelona, Spain) shown in Figure 2a. The yarn was constructed using an eight needle cylinder having a diameter of 4 mm. Eight 167dtex/48 filament (1/167/48) undyed polyester yarns (J.H.Ashworth and son, Cheshire, UK) were utilised for the eight needles and used as covering yarns. Six other packing yarns (1/167/48 polyester yarns) were used along with the carrier yarn in the core. The packing yarns provided padding for the sensor. Henceforth, this yarn will be referred to as the knit-braided yarn. The next method utilised a Herzog^®^ RU 1/24-80 braiding machine (Oldenburg, Germany), displayed in Figure 2b, to interlace 24 bobbins of the 1/167/48 polyester yarn at a braiding lay length of five, to create the braided yarn. The carrier yarn containing the flexible sensors was sent through the core and the polyester yarns were interlaced around it. The last method utilised was a double cover technique using a Menegatto 1500 (Menegatto S.r.L, Molgora, Italy) presented in Figure 2c. The machine used two 1/167/48 polyester yarns on the top and bottom cover of the machine. The top cover was set to 1500 twists per meter (TPM) and the bottom cover was set to 1800 TPM. This yarn will be referred to as the double-covered yarn.

### 2.3. Measuring the Temperature Coefficient of Resistance

Skin temperature ranges from 20 °C to 40 °C during daily activities [33]. Therefore, it is vital to identify the sensitivity of the temperature-sensing yarns for this temperature range. The RTDs, when embedded within a textile yarn, are not in direct contact with the surface being measured. Literature has shown alterations in the effective sensitivity of rigid thermistors incorporated within textile yarns [29]. In these experiments, we investigate the impacts of the textile filaments and the yarn fabrication techniques on the effective sensitivity of textile-embedded flexible RTD.

For the experiments, an EchoTherm™ IC50 digital Chilling/Heating Dry Bath (Torrey Pines Scientific Inc., Carlsbad, CA, USA), henceforth referred to as hot plate, was utilised to simulate skin temperature. The hotplate had an accuracy of ±0.2 °C. Even though the heat conductivity of the hot plate was different to that of human skin, this was one of the most accurate methods to characterise these sensors. The flexible RTDs were attached onto the surface of the hot plate. Copper wires were soldered to the RTDs to measure its resistance while on the hot plate. Thereafter, the exact same experiment was conducted for the RTDs embedded in sensing yarns. The temperature of the plate was increased from 20 °C to 40 °C in increments of 2 °C every 3 min. For each increment, a time delay of 2.5 min was imposed to allow the sensor to reach a steady-state. This was regarded acceptable since 2.5 min was significantly longer than the thermal time constant measured in Section 3.3. Afterwards, 30 measurements were taken at one-second intervals using a Keysight 34465A digital multimeter (Santa Rosa, CA, USA). The average resistance was then calculated from these 30 measurements. This method ensured that all the measurements were taken once the sensor reached thermal equilibrium. For all experiments, the RTDs were characterised individually.

### 2.4. Response Time Experiments

Temperature-sensing yarns must have a faster response time compared to the rate of change in skin temperature. This is necessary in order to acquire accurate and continuous temperature measurements. In general, skin temperature changes take place over a time span of several minutes [5,6]. Typical temperature sensors have fast response times <30 s [34,35]. However, since the RTDs positioned in the yarn are not in direct contact with the surface being measured, the heat needs to be transferred through the polyester filaments which provide thermal resistance and restricts the flow of heat. For this reason, it is particularly important to measure their response time. Previous studies have shown that embedding rigid thermistors within yarns impacts their response when subjected to heating and cooling [36]. Here, the response time of the flexible RTDs before and after they were embedded within the yarn was assessed, in order to understand the impact of yarn filaments and the yarn manufacturing technique.

As the human skin temperature can rise as high as 40 °C [33,37], this value was chosen as the hot plate temperature to investigate the performance of the sensors. Initially, the samples were left at room temperature (22.9 ± 1.1 °C) for 3 min. Then, the samples were attached onto the surface of the hotplate which was set to 40 °C and left for a further 3 min. Finally, the sensors were detached from the hotplate and left to cool down to room temperature for another 3 min. These conditions were chosen to depict the human skin and ambient temperatures. The temperature of the laboratory was monitored during the experiments using a K-Type thermocouple connected to a Digitron 2022T digital thermometer (Port Talbot, UK). The resistance measurements from the RTDs were obtained every 0.5 s. Each RTD yarn was measured three times and the average was calculated. To obtain the response time for each type of sensing yarn, the average from three samples were taken.

### 2.5. Temperature Cyclic Test

The temperature sensors, when worn in a textile, would have to provide continuous and repeatable temperature measurements. Therefore, a cyclic test was conducted on the yarns, where the yarns were attached to the hot plate and the temperature of the hot plate was cycled from 20 °C to 40 °C for 5 cycles. Each step was kept for 5 min and the resistance measurements were captured every second.

### 2.6. Bending Experiments Conducted on the Sensing Yarns

Yarns, when manufactured into textiles, undergo several cycles of bending. When the flexible RTD is bent, it induces a strain on the surface of the thin active metal layer. This can lead to a rupture or a change in resistance as a result of the strain [38,39]. Therefore, the following experiments were conducted to identify the effects of bending on the performance of the yarns. Initially, the temperature-sensing yarns were kept on the hot plate set to 40 °C for 3 min. Afterwards, they were removed from the hot plate and underwent 100 bend/flat cycles around a 25 mm cylinder. Finally, the temperature sensor was reattached onto the hot plate for a further 3 min. The temperature measured between 2.5 min to 3 min after the yarns were positioned on the hotplate before and after bending was utilised to identify any changes in resistance. The 25 mm diameter was considered adequate since this roughly corresponds to the smallest bending radii in a human body (i.e., diameter of a finger).

The strain on the metal surface of the thin-film sensor due to this bending radius of 25 mm was calculated as 0.094%, 0.099%, and 0.099% for the knit braided, braided, and double covered yarns, respectively. The strain was calculated using the following equation [38,39].

(1) $$\epsilon_{top}=\left(d_{f}+d_{s}\right)/2R,$$

where, $\epsilon_{top}$ is the strain at the metal surface, $d_{f}$ is the metal layer thickness and $d_{s}$ is the thickness of the substrate, and R is the radius of curvature. For calculation purposes, we assumed that the flexible strip was positioned at the centre of the sensing yarns. This equation was utilised since the substrate thickness was significantly greater than the metal layer thickness.

### 2.7. Manufacturing of a Prototype Armband to Measure Changes in Skin Temperature

A prototype armband was fabricated to demonstrate the possibility of creating a textile garment incorporated with these yarns to record changes in skin temperature. The armband was knitted on a Stoll ADF-3 E18 machine (Stoll, Reutlingen, Germany) using 2/333 dtex (1ply) merino wool yarns (Uppingham yarns, Uppingham, UK). The armband was constructed with an all needle interlock structure with a tubular structure to insert the yarns positioned in the centre. For this armband, the braided sensing yarn was inserted into the tubular structure. This technique of fabricating textiles with predefined channels for incorporating sensing yarns has numerous advantages. These include the precise positioning of the sensing areas of the yarns and the ability to remove the sensing yarns from the textile if the yarn malfunctions or during the recycling of the textile [40].

A user trial was conducted utilising the armband to measure temperature of the upper arm. Skin temperature of the upper arm is an indicator for exercise performance [11,12,13]. Initially, the armband was not worn and left at room temperature for 5 min. Afterwards, the armband was worn on the arm and the measurements were obtained for the next 5 min. Then, the armband was taken off and kept at room temperature again for a further 5 min. During this time, the wearer performed bicep curls using a weight of 10 kg. The arm band was taken off during the exercise task to eliminate measurement errors caused by movement artefacts and contact issues between the sensor and the skin. In the future, these issues could be overcome by using a well fitted armband composed of elastane [40]. The armband was then wrapped around the upper arm and the temperature was measured for 5 min. Then, the experiment was repeated for the second time. A K-Type thermocouple connected to a digitron 2022T digital thermometer was utilised to monitor room temperature and skin temperature. When measuring skin temperature, the thermocouple was positioned in between the armband and the arm, just below the position of the temperature-sensing yarn. The results are presented in the next section.

## 3. Results

The fabricated temperature-sensing yarns and their performance with regard to the experiments described in the previous section are given here. The results for the skin-temperature-measuring armband is also presented.

### 3.1. Temperature-Sensing Yarn

**Figure 3:**
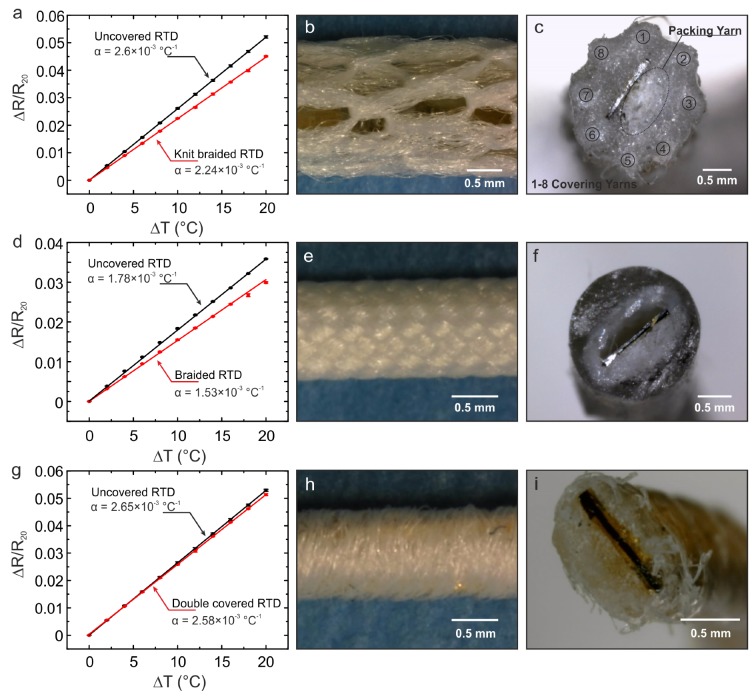
Change in resistance as a response to the change in temperature before and after the RTDs were covered using the three different covering techniques. A linear fitting was applied to calculate α. (**a**) Measurements from the RTD positioned within a knit-braided structure. The RTD incorporated in the structure has an effective α of 2240 ± 6 × 10^−6^ °C^−1^ compared to an α of 2600 ± 4 × 10^−6^ °C^−1^ when it was not covered. (**b**) Close-up image of the knit-braided yarn obtained using a Dino-Lite premier digital microscope (New Taipei City, Taiwan). (**c**) Cross section of the knit braided yarn captured using the digital microscope. (**d**) The RTD positioned in the braided yarn has an α of 1530 ± 8 × 10^−6^ °C^−1^ compared to the uncovered RTD’s α 1780 ± 7 × 10^−6^ °C^−1^. (**e**) Close up of the braided yarn. (**f**) Cross section of the braided yarn obtained using the digital microscope. (**g**) The RTD covered using the double covering technique has an α of 2580 ± 1 × 10^−6^ °C^−1^ when compared to 2650 ± 8 × 10^−6^ °C^−1^ when it was uncovered. (**h**) The double-covered yarn close up and the cross section of it shown in (**i**).

The flexible RTDs were successfully integrated within the textile yarns utilising all three of the yarn covering techniques (knit braiding, braiding, and covering). The knit braided yarn had a diameter of 3.40 ± 0.07 mm. The braided and the double-covered yarn had flat topologies, where the braided one had a width of 1.10 ± 0.03 mm and a thickness of 0.73 ± 0.04 mm; the double-covered one had a width of 1.05 ± 0.06 mm and a thickness of 0.51 ± 0.04 mm, where the RTDs were positioned. Upon visual inspection, the braided RTD had the best textile cover (Figure 3e) when compared to the rest (Figure 3b,h). Nevertheless, the double-covered yarn had the smallest dimensions.

### 3.2. Temperature Coefficient of Resistance of the Temperature Sensing Yarns

In RTDs, the temperature coefficient of resistance is measured to predict the sensitivity of the sensor. The resistance of metals vary linearly with temperature. This can be obtained using the following equation:

(2) $$R_{t}=R_{ref}\left(1+\alpha \left(T-T_{ref}\right)\right),$$

where *R_t_* is the resistance at temperature *T* in degrees Celsius, *R_ref_* is the reference resistance at 20 °C, α is the temperature coefficient of resistance, and *T_ref_* is the reference temperature (20 °C) at which α is specified [41,42].

The flexible RTDs had a *R_ref_* of 201.31 ± 7.56 Ω at 20 °C which was significantly larger than the resistance of the interconnects (i.e., solder joint and the copper wire) which came to 1.74 ± 0.18 Ω. The temperature sensitivities of all the sensors were slightly different due to process variations, therefore they were individually calibrated. Hence, for all experiments, the changes in effective sensitivity was assessed individually for each RTD before and after it was covered.

It can be observed from the results in Figure 3 that all the yarns respond linearly to changes in temperature. The α from the graphs were obtained by calculating the gradient of the best fit line. The effective sensitivity of the yarns was lower when compared to the uncovered RTDs. This occurs due to the sensor not being in direct contact with the surface being measured. Therefore, the sensors are not measuring the temperature of the plate, but the temperature within the core of the yarn. Nevertheless, the maximum decrease in effective sensitivity as a result of the different covering techniques was only 14%, which was observed in the knit-braided yarn. Table 1 compares the α for each sensing yarn fabrication technique. The double-covered yarn has the lowest reduction in α compared to the rest.

### 3.3. Response Time of the Temperature-Sensing Yarns

**Figure 4:**
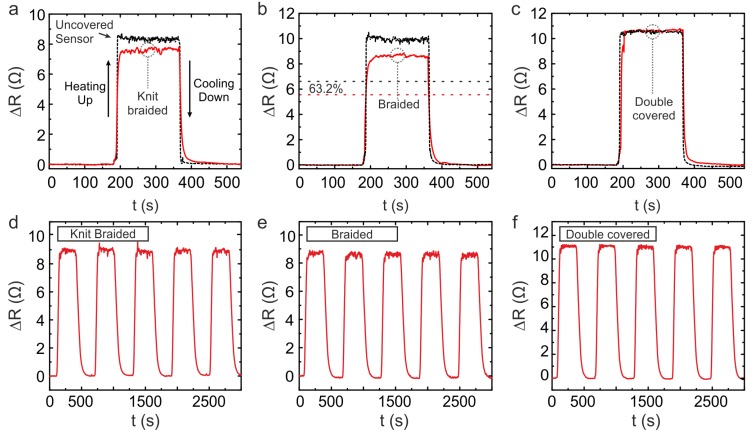
Response time and cyclic experiments for the yarns. For response time experiments, a representative comparison before and after embedding the thin-film temperature sensors into the yarns is presented. The measurements of response time from the knit-braided, braided, and double-covered yarns before and after they were covered are shown in (**a**–**c**), respectively. The thermal time constant corresponds to the time taken by the sensor’s resistance to reach 63.2% of the final value once a sensor is subjected to a step change from ambient temperature to 40.0 °C and vice versa. This is indicated by the horizontal dashed lines in (**b**) which correspond to 63.2% of the maximum value. Finally, (**d**–**f**) are the results from the cyclic tests conducted on the knit-braided, braided, and double-covered yarns, respectively, where the temperature was cycled from 20.0 °C to 40.0 °C for 5 cycles.

The time taken for a sensor to heat and cool can be evaluated by looking at the sensors thermal time constant [43]. The thermal time constant (τ) is defined as the time taken by a temperature sensor to reach 63.2% of its final temperature when subjected to a step change in temperature [43]. Figure 4 displays the response of three RTDs before and after they were embedded within different yarn structures.

The effect on the thermal time constant for heating and cooling as a result of including the RTD within a textile yarn is illustrated in Table 2. It can be observed that incorporating the RTD within the yarn has increased τ as a result of the sensing area of the RTD not being in direct contact with the surface being measured. Furthermore, the added polyester fibres also increases the heat capacity. Nevertheless, in all cases, τ was <10 s across all samples. The RTD embedded within the double-covered structure showed the fastest τ in response to heating and cooling when compared to the other two.

### 3.4. Temperature Cyclic Tests Results

These sensing yarns must be able to continuously produce repeatable measurements when they are utilised for monitoring skin temperature. Figure 4c–e display the thermal cyclic measurements obtained from the three sensing yarns. It is illustrated that all the temperature-sensing yarns provide repeatable measurements.

### 3.5. Results of Bending Experiments on the Temperature-Sensing Yarns

**Figure 5:**
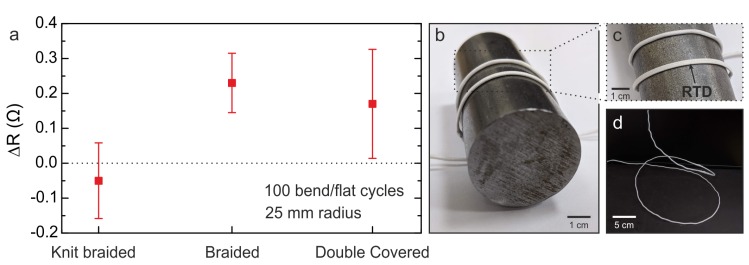
Bending experiments. (**a**) Resistance drift of the three sensing yarns due to 100 bending cycles. The resistance was measured when the yarn was flat on the hot plate at 40.0 °C before and after the cyclic test. (**b**) The braided sensing yarn wrapped around the 25 mm diameter cylinder. (**c**) Close-up image of the braided sensing yarn conforming around the cylinder. (**d**) An image of a twisted and coiled braided sensing yarn.

Temperature measurements from all three yarns were not affected by bending over the 25 mm diameter cylinder. The results are summarized in Figure 5. The resistance change was <0.2% for all the three yarns (Figure 5a). Therefore, it can be stated that 100 bending cycles over a 25 mm diameter cylinder had no impact on the sensing yarn measurements. Thus, strains of 0.094%, 0.099%, and 0.099% had no impact on the measurements from the sensing yarns.

### 3.6. Temperature Measurements for the Armband Integrated with the Temperature-Sensing Yarn

**Figure 6:**
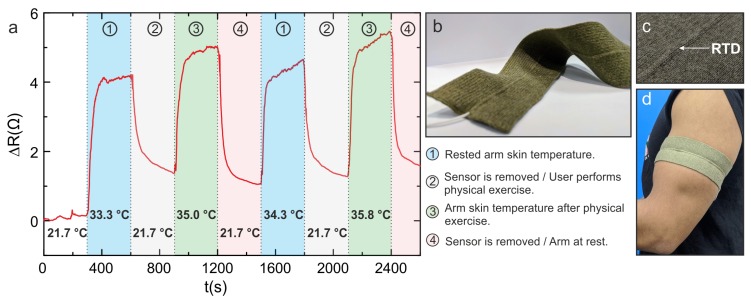
(**a**) Temperature measurements obtained from the temperature sensing yarn positioned within the armband during the preliminary user trial. (**b**) The prototype armband with the temperature-sensing yarn positioned in the centre. (**c**) Close up image of the sensing area of the armband. (**d**) The armband worn on the upper arm.

This experiment was set up to determine the yarn’s ability to measure variation in skin temperature during a physical activity. The measurements of the armband are shown in Figure 6a. The results show that the resistance increases as soon as the armband is worn. Nevertheless, it does not return to its original resistance when the armband is taken off and left at room temperature. This is most likely due to the thermal inertia of all the materials surrounding the sensor and due to the low thermal conductivity of the wool armband which retains the heat of the body. Figure 6a also displays the increase in temperature as a result of the physical activity when compared to when the arm was left to rest. This pattern repeats on the second set of experiments. The results show an adequate performance of the sensing yarns for measuring skin temperature in sport applications.

## 4. Discussion

Three different covering techniques were successfully utilised to cover the flexible RTDs. One limitation with the fabrication method of temperature-sensing yarns shown here, was the dimensions of the solder joint that connected the strip onto copper wires. The solder joint increased the size of the carrier yarn which hindered and obstructed the flow of these yarns within the interior of the industrial machines. This in turn damaged the solder joints of the sensors which led to the production of limited samples. In addition, the solder joints also increased the size of the final sensing yarns. One method of addressing this is by utilising a DC welder to weld the wire onto the metal contacts of the Kapton strip. This would reduce the size of the interconnecting joint and therefore would decrease the thickness and width of the resulting yarn. For this work, we manufactured three samples for each covering technique. Due to damages in the solder joints as a result of the manufacturing process, there were large variations in $R_{ref}$. Therefore, one representative sample for each covering technique was analysed for the temperature coefficient of resistance, cyclic, and bending experiments, whereas all the nine samples were utilised for the response time experiment. In regard to the performance of the sensors, it was observed that the covered sensors demonstrated lower effective sensitivities when compared to the uncovered sensors. The reduction in sensitivities for the RTDs embedded in knit-braided, braided, and double-covered structures was recorded as 2.5 × 10^−4^ °C^−1^, 3.6× 10^−4^ °C^−1^, and 7× 10^−5^ °C^−1^, respectively. Nevertheless, the sensitivity of the covered sensors remained linear and the maximum reduction in effective sensitivity was only 14%. In addition, the effects of the different yarn fabrication techniques on the response and recovery time of the sensors were observed to be insignificant when compared to the rate of change in skin temperature. The thermal time constants for all the yarns was <10 s for heating and cooling. All three yarns produced repeatable measurements and were not effected by unidirectional bending. The fabrication techniques of the sensing yarns and the manufacturing process of the armband did not require the RTDs to undergo other forms of bending such as lateral twisting. However, this needs to assessed in the future if these yarns are integrated into textiles using different manufacturing techniques.

The double-covered yarn demonstrated a superior performance when compared to the rest. The reduction in effective α was lowest for the double-covered yarn when compared to the braided and knit-braided yarns. The double-covered yarn also demonstrated a faster response to step changes in temperature when compared to the other two yarns. This is due to the smaller thickness of the double-covered structure, which in turn positions the RTD closer to the surface being measured. This effect can be related to Fourier’s law of heat conduction, where the decrease in measured temperature is directly proportional to the distance between the sensing area and the surface being measured. This causes the reduction in effective sensitivity of the RTDs. Another reason for the reduction in effective sensitivity is the presence of air in between the sensor and the covering yarns. Air is a good thermal insulator which introduces a thermal resistance in between the surface being measured and the RTD. The knit-braided yarn had a larger diameter and a loose fibre cover with plenty of space for air compared to the other two. This resulted in a higher reduction in effective sensitivity. Hence, to minimize the reduction in effective sensitivity, the covering yarns need to fit tightly around the sensor.

The results from the armband demonstrate that the sensing yarns could be utilised for measuring changes in skin temperature. Nevertheless, in order to capture accurate skin temperature, these yarns need to be calibrated. In order to ensure a good performance is achieved from these temperature sensing yarns, the thickness of the textile cover needs to be at a minimum and the cover needs to tightly fit around the sensor. The knit braider is limited in its ability to provide yarn covers that tightly fit the dimensions of the sensors due to its predefined cylinder sizes, which results in creating yarns with a preset diameter. Therefore, the braided and the double covered techniques are the most suitable for creating tighter textile covers for these flexible sensors. Additionally, the parameters of the braiding and double covering machines could be optimised further to achieve better fibre covers. This can be obtained by altering the lay length in the braiding machine or by changing the number of twists per meter in the top and bottom covers of the double cover machine. Another important aspect that needs to be considered when manufacturing electronic textiles is the washability of these garments and the impact of dirt and sweat on the performance of the sensors. These yarns can be fabricated in a washable manner by encapsulating the flexible RTDs in a hydrophobic polydimethylsiloxane layer before they are incorporated within the yarn [31,32]. The enamelled copper wires used in this work should prevent short circuits from forming due to sweat. This needs to be assessed in future work. Once improved, these yarns could be used to knit or weave any piece of textile clothing.

## 5. Conclusions

This paper demonstrates an unobtrusive integration method for flexible sensors within a textile. Flexible RTDs were successfully integrated within textile yarns using three common yarn fabrication techniques, specifically, knit braiding, braiding, and double covering. The sensitivities of the RTDs remained linear after they were incorporated within the textile yarns. However, the effective sensitivity of the integrated RTDs were lower than the uncovered RTDs. This is a result of the sensing area of the RTD not being in direct contact with the surface being measured and due to the textiles’ thermal insulation properties. Nevertheless, in all three sensing yarns, the maximum percentage reduction in effective sensitivity was only 14%. Concurrently, the thermal time constant for heating and cooling increased as a result of the textile cover, but in all yarns, the thermal time constant for both heating and cooling was less than 10 s. This can be regarded as a fast response since it was well within the temporal resolution required for detecting skin temperature changes, which in general takes a few minutes [5,6]. The yarns demonstrated repeatable measurements and they were not effected by bending. The double-covered yarns demonstrated a better performance when compared to the other two. This was due to its smaller size and tight cover around the flexible sensor, which led to the sensing area of the RTD being closer to the surface. Finally, the temperature measurements from the armband demonstrated that these sensing yarns could be used to manufacture textile garments that measure changes in skin temperature. This technique of fabricating electronic textiles could be industrialised in the future, utilising roll-to-roll fabrication for the sensing elements and using these industrial yarn covering machines for the fabrication of the sensing yarns. Once calibrated, these yarns can be utilised to manufacture textiles that could be used for applications such as thermal discomfort detection in prosthetic socks, socks for early prediction of diabetic foot ulcers, textiles to continuously measure temperature of infants, and bandages to monitor infections in wounds.

## Figures and Tables

**Table 1:** Influence of different embedding methods on the temperature coefficient of resistance.

Method of	Uncovered	Covered	Decrease	Percentage	Room
Embedding	RTD α	RTD α	in α	Decrease in α	Temperature
	(°C^−1^)	(°C^−1^)	(°C^−1^)	(%)	(°C^−1^)
Knit braid	2.60 × 10^−3^	2.24 × 10^−3^	3.6 × 10^−4^	13.8	24.3
Braid	1.78 × 10^−3^	1.53 × 10^−3^	2.5 × 10^−4^	14.0	24.0
Double cover	2.65 × 10^−3^	2.58 × 10^−3^	7 × 10^−5^	2.6	20.9

**Table 2:** Effects of different yarn fabrication techniques on the thermal time constant.

Method of Embedding	τ for Heating	τ for Cooling	Increase in τ for Heating	Increase in τ for Cooling
	(s)	(s)	(s)	(s)
Knit braid	4.7 ± 0.5	6.2 ± 1.0	3.3 ± 0.5	4.5 ± 1.0
Braid	3.4 ± 0.9	5 ± 0.6	1.9 ± 1.0	3.1 ± 0.9
Double cover	2.4 ± 0.5	3.3 ± 0.6	1.1 ± 0.7	1.8 ± 0.6
